# Ten simple rules to colorize biological data visualization

**DOI:** 10.1371/journal.pcbi.1008259

**Published:** 2020-10-15

**Authors:** Georges Hattab, Theresa-Marie Rhyne, Dominik Heider

**Affiliations:** 1 Department of Mathematics and Computer Science, University of Marburg, Marburg, Germany; 2 Durham, North Carolina, United States of America; Dassault Systemes BIOVIA, UNITED STATES

Methods for visualization of biological data continue to improve, but there is still a fundamental challenge in colorization of these visualizations (vis). Visual representation of biological data should not overwhelm, obscure, or bias the findings, but rather make them more understandable. This is often due to the challenge of how to use color effectively in creating visualizations. The recent global adoption of data vis has helped address this challenge in some fields, but it remains open in the biological domain. The visualization of biological data deals with the application of computer graphics, scientific visualization, and information visualization in various areas of the life sciences. This paper describes 10 simple rules to colorize biological data visualization.

Rule 1: Identify the nature of your data
Rule 2: Select a color space
Rule 3: Create a color palette based on the selected color space
Rule 4: Apply the color palette to your data set for visualization
Rule 5: Check for color context in your data vis after the color palette is applied
Rule 6: Evaluate interactions of colors in your data visualization
Rule 7: Be aware of color conventions and definitions in your particular discipline
Rule 8: Assess color deficiencies
Rule 9: Consider web content accessibility and print realities
Rule 10: Get it right in black and white

## Rule 1: Identify the nature of your data

Data are valuable records of information. Visualizing data is an important and powerful way of relating the ideas, experiences, and stories contained in these data. Graphics and data visualization facilitate the presentation and communication of biological information in diverse contexts, shaping narratives, ideas, and experiences. To give shape to the information contained within your data, knowing the nature of the data is important. Borrowing terms from the domain knowledge of descriptive statistics, data such as gender, age, height, weight, and eye color are referred to as variables. A variable is simply defined as what is being observed. The type of a variable relates to the nature of the data.

One way to differentiate between types of variables is to rely on the nature of the information within the values assigned to a variable. This is known as the level or scale of measurement and classifies the observed variables into 4 levels: nominal, ordinal, interval, and ratio [1]. These can also be assigned to 2 separate data kinds: qualitative or categorical (nominal, ordinal) and quantitative (interval, ratio). In the following list, we describe and explain each with 1 example:

- The **Nominal** level describes attributes of a variable differentiated only by name (category), and there is no order (rank, direction, or position).

Example: Gender, biological species, the eye color (blue, green, brown, etc.), the domain taxonomic rank (archaea, bacteria, and eukarya), the blood type (A, B, AB, O), the type of bacteria (coccus, bacillus, spirillum, etc.). They are a multivalued variable, and there’s no clear scale on which to fit the different values.

- The **Ordinal** level describes categorical attributes of a variable differentiated by order (rank, scale, or position), yet there is no information on the relative degree of difference among them. Be careful; such a variable may be coded numerically.

Example: Heat (low, medium, high); severity of a disease (mild, moderate, severe); an agreement scale, e.g., Likert scale, (strongly disagree, disagree, no opinion, agree, or strongly agree).

- The **Interval** level describes attributes of a variable differentiated by the degree of difference between them, without an absolute zero and without a known ratio among the attributes. It is typical that this variable has numerical values that are positive, negative, or zero.

Example: The metric Celsius temperature scale, the temperature difference (Celsius and Kelvin), the interval of 1 calendar year. The difference between 20° and 30°C is the same as the difference between 25° and 35°C.

- The **ratio** level describes attributes of a variable differentiated by the degree of difference among them, with an absolute zero and with a known ratio among the attributes. It is atypical to have negative values, because that would indicate less than nothing.

Example: Age, height, mass or weight, duration, the Kelvin temperature scale.

Moreover, quantitative data (interval or ratio) that assume numeric values can be further classified as either discrete or continuous.

- **Discrete** (countable) variables assume only whole numbers and some kind of count.

Example: Age and Date are discrete. While Age stays constant for a period of 1 year, Date does for 24 hours. They both jump or increase by 1.

- **Continuous** (any value in a defined range) variables can take any value in some range of values. The observation of such a measurement is limited by the measuring instruments. Units are often reported.

Example: Height (cm, in), weight (kg, pounds), temperature (°C, Fahrenheit), and time (h, min, s). Temperature increases gradually and time flows continuously.

The **binary** or **dichotomous** variable type is a special type, when there are only 2 possible values. Example: Yes or No questionnaire and the binary digit (0 or 1). Table 1 introduces the 4 levels of measurements according to 4 different measurement-related classes, including the resolution of the measurement from lowest to highest.

**Table 1:** The 4 levels of measurements. The levels are compared using 4 measurement-related classes: resolution, property, mathematical operators, and central tendency [2].

Level	Measurement resolution	Measure property	Mathematical operators	Central tendency
Nominal	Lowest	Classification, membership	=, ≠	Mode
Ordinal	Low	Comparison, level	>, <	Median
Interval	High	Difference, affinity	+, -	Mean, deviation, variance
Ratio	Highest	Magnitude, amount	×, /	Geometric mean, coefficient of variation

Before proceeding to Rule 2, list all the relevant variables in your data set, and identify their types.

## Rule 2: Select a color space

A color space refers to a color model in which colors turn into numbers. Based on a set of primary colors, a color model creates many colors. Each model has a specific range of colors it can produce, which defines the color space. Generally, red, green, and blue (RGB) and cyan, magenta, yellow, and black (CMYK) are the most common systems (c.f., Rule 9), but there are others [3]. For example, the hue, saturation, and brightness/value (HSB/HSV) color spaces are alternative representations of the RGB color model or standard red green blue (sRGB) color space [4,5]. For further information on these dimensions, the website of David Briggs entitled The Dimensions of Color is a trove of information on color theory and usage: huevaluechroma.com.

Traditional color tools, e.g., color wheel, encourage artistic/manual color selection. Numeric values for color or code are different because we are encouraged to think about colors as numbers in a specific color space. Moreover, since discrepancies may arise between the numbers we choose and the output color, color spaces should be perceptually uniform. In the field of color science, efforts have been made to build color spaces that are independent of particular color display or reproduction devices. One of the first ones developed by the International Commission on Illumination (CIE) was the CIE 1931 XYZ color space. In 1942, MacAdam published color perception tests with humans that showed the CIE 1931 XYZ color space to be nonperceptually uniform [6]. A color space is perceptually uniform, when a change of length x in any direction of the color space is perceived by a human as the same change. As a result, efforts were made to create perceptually uniform color spaces. CIE Luv and CIE Lab were approved in 1976 as respectable attempts to address this issue. We recommend using CIE Luv and CIE Lab color spaces or other advanced color appearance models in your work. Fairchild's book on "Color Appearance Models" develops these concepts further if you desire to read further on this subject [7]. The motivation behind these color spaces is to closely align the space to how human vision perceives color attributes.

To this end, we first present color spaces that are commonly used (c.f., Table 2), then discuss those that address the problem of perceptual uniformity. Thanks to sophisticated color transformations, the dimensions in which light mixes reflect how human vision works. We report on various characteristics that need to be considered and briefly explain them: model, linear, intuitive, component separation, and device-dependent.

- **Model**: an orderly system for creating a complete range of colors from a small set of primary colors
- **Linear**: a change of the same amount in a color value should produce a change of about the same visual importance
- **Intuitive**: refers to the easy-to-remap property of color dimensions into different color models
- **Component separation**: refers to separating 1 color dimension relative to other dimensions. For example, hue, saturation, and lightness (HSL) separates the luminance component (luma) and is particularly useful in the domain knowledge of image processing
- **Device-dependent**: the condition that the color space relies on the used equipment to set up, produce, and render it.

**Table 2:** Pros and cons of commonly used color spaces.

Color space	Model	Linear	Intuitive	Component separation	Device-dependent
RGB	Additive	0	Low		Yes
CMYK	Subtractive	0	Low		Yes
HSL	Transform	0	High	Luminance	Yes
HSB/HSV	Transform	0	High	Luminance, chroma	Yes
LAB/LUV	Additive/translational	1	Moderate	Luminance, chroma	No

CIE LAB/LUV, lightness, a:/u: greenish, b: brightness/v; bluish; CMYK, cyan, magenta, yellow, black; RGB, red, green, blue; HSB/HSV, hue, saturation, brightness/value; HSL, hue, saturation, lightness.

Luminance is the visible energy of light or the physical light energy weighted according to the wavelength-by-wavelength response of the human visual system (CIE e-ILV 17-711). Chroma is the colorfulness of an area judged as a proportion of the brightness of a similarly illuminated area that appears white or highly transmitting (CIE, 2011, 17-139). While chromaticity describes the psychophysical color of a light, it is independent of its intensity (luminance).

The listed perceptually uniform color spaces are superior to the RGB and CMYK color spaces. RGB is accepted to represent colors, yet it is inadequate for color processing and is not the industry standard. Since CMYK is mostly used in print, it has many disadvantages and is further discussed under Rule 9. However, they are not without confounding effects such as brightness changing dramatically with hue (i.e., HSL and HSB/HSV). Both LUV and LAB aspire to perceptual uniformity. While both have been adopted by CIE, relative perceptual differences of a set of colors in the LAB space can be observed by relying on its 3 components and calculating the Euclidean distance among said colors [8]. Since they are device independent, we suggest using either color spaces. Provided one is selected, we now need to create a suitable color palette for the data.

## Rule 3: Create a color palette based on the selected color space

Creating a color palette is much like choosing an outfit. Contrary to the proverb “Clothes don't make the monk,” it’s important to know the rules that permit the selection of colors to colorize your data visualization. To select a color palette based on a specific color space, a color wheel is often used. It is a tool that organizes different colors around a circle to show the relationship between the colors. Typically, the color wheel contains 12 colors. Newton is credited with creating the first color wheel when he closed the linear color spectrum into a color circle [9]. Newton's wheel was published in the early 1700s after researching the concepts in the late 1600s. Over the centuries, artists and color scientists amplified his concept to include color harmonies. Creating a color harmony is the process of choosing colors that work well together in the composition of an image. Similar to concepts in music, these harmonies are based around color combinations on the color wheel that help to provide common guidelines for how color hues will work together.

**Fig 1:**
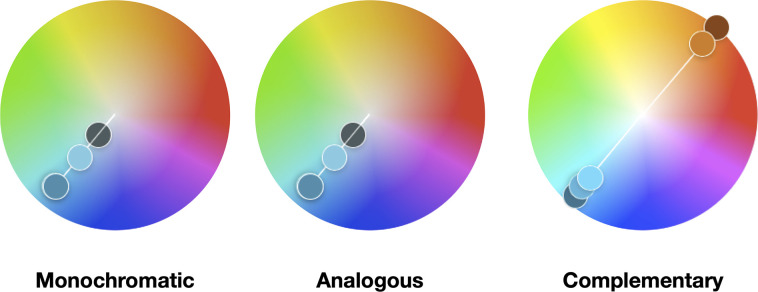
Example of 3 color harmonies in the key of cyan. These harmonies were created using the Adobe Color web tool (color.adobe.com). They are color blind friendly palettes and are presented in Web Hex format. Monochromatic: 2C7C9D, 65BFDA, 39484C. Analogous: 5FE896, 5FF3E3, 3CA7D2, 1E78EF, 1938E3. Complementary: 22607C, 3CA6D0, 4CCFFA, D06D21, 7B3514.

We can distinguish software and/or web tools that help to create color schemes using the color wheel, namely Adobe Color (Adobe Inc., San Jose, California, United States; color.adobe.com) and Paletton–The Color Scheme Designer (Petr Staníček, Prague, Czech Republic; paletton.com). Apart from creating aesthetically pleasing color combinations, harmonies can be used to guide the creation of color palettes. They include monochromatic, analogous, and complementary [10–12]. Fig 1 depicts 3 examples of harmonies in the key of cyan. To observe the harmonies, one should focus on the small dots of color to trace a particular hue arrangement (monochrome, analogous, and complementary).

- **Monochromatic** or monotone chromatic is one single hue and its variations in terms of tints, shades, and saturation. A particular example is a monotone scheme, yet achromatic (without a hue), consisting of only gray values from black to white, i.e., gray scale.
- **Analogous** colors are those that lie on either side of any given color or are separated by one. Often these are color schemes found in nature.
- **Complementary** colors are the colors which are directly opposite from one another on the color wheel. They often contrast and stand out against each other. They are useful when used as the highlight colors in the data.

**Fig 2:**
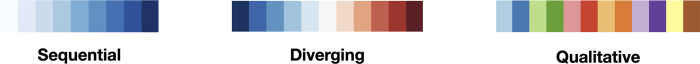
Example of 3 color palettes according to the 3 main data types. These color palettes are based on the ColorBrewer schemes. They comprise different classes and are given specific names in ColorBrewer: sequential (9-class Blues), diverging (11-class RdBu), and qualitative (12-class Paired).

To better align color usage to data types, information designers and data scientists simplify the aforementioned data types (c.f., Rule 1) to 3 main types: sequential, diverging, and qualitative. These classifications were developed in the ColorBrewer tool (Cynthia Brewer, Mark Harrower, and The Pennsylvania State University, State College, Pennsylvania, United States), initially designed to provide color advice for cartography [13,14]. The concept has since been adopted by the data visualization community as reflected in Munzner’s Visualization Analysis and Design textbook [15]. The web tool ColorBrewer can be found at colorbrewer2.org. Fig 2 illustrates an example of color palette for each data type.

- **Sequential** palettes are suitable for ordered data that varies from low to high values. Depending on which side is most important to the viewer, the visual encoding is a variation between 2 colors that range from white or a lighter color to black or a darker color, respectively. This color usage is a lightness stepwise variation with typically important data values having darker colors. These palettes correspond to monochromatic color palettes that comprise variations of 1 color.
- **Diverging** palettes show visual variation in 2 directions. Mostly used to put equal emphasis on midrange values and extreme values at both ends of interval data ranges, they are typically symmetrical. Colors increase in darkness to represent differences around a break point (e.g., zero-change or mean value) from a specific meaningful midrange value in the data.
- **Qualitative** palettes do not rely or imply on magnitude differences among classes. Typically, hues are employed with consistent lightness to represent nominal and categorical data. There are 2 additional variants: paired and accent. Although dealing with nonordered data, while paired palettes deal with pairs by visually relating classes, accent palettes deal with accentuating relevant classes with more saturated colors.

There are many works in the literature that guide the user to create color palettes that are fitting in the large space of possible color palettes or colormaps [16,17]. Apart from the aforementioned tool ColorBrewer, we mention 2 palettes: 1 for sequential and the other for qualitative data. For sequential data, the viridis palette is notable [18]. It is perceptually uniform and displays monotonically increasing luminance in multiple hues. Thanks to the viridis palette, and other palettes (e.g., magma, inferno), all data points of a sequential data set have equal visual importance. Moreover, as we will see it later in Rule 8, these palettes are friendly to color deficiencies and color blindness. For qualitative data, the Tableau 10 color palette is notable [19]. It contains several very distinct hues with a range of lightness value. Although it is designed with 10 colors, and it fairs well for the Trichromacy anomalies, the usage of all its colors is a challenge for other color deficiencies. We discuss the limitations of color for qualitative data in Rules 4 and 8.

For the sake of completeness, we also would like to mention 1 additional web tool to create color palettes, namely Colorgorical (Connor Gramazio, with advisement from David Laidlaw and Karen Schloss at Brown University, Providence, Rhode Island, United States). It is available at http://vrl.cs.brown.edu/color [20].

## Rule 4: Apply the color palette to your data set for visualization

To apply a selected color palette, one needs to consider the process of mapping color to data points. Based on Rule 3, we consider the 3 different palettes for color mapping: sequential, diverging, and qualitative.

**Fig 3:**
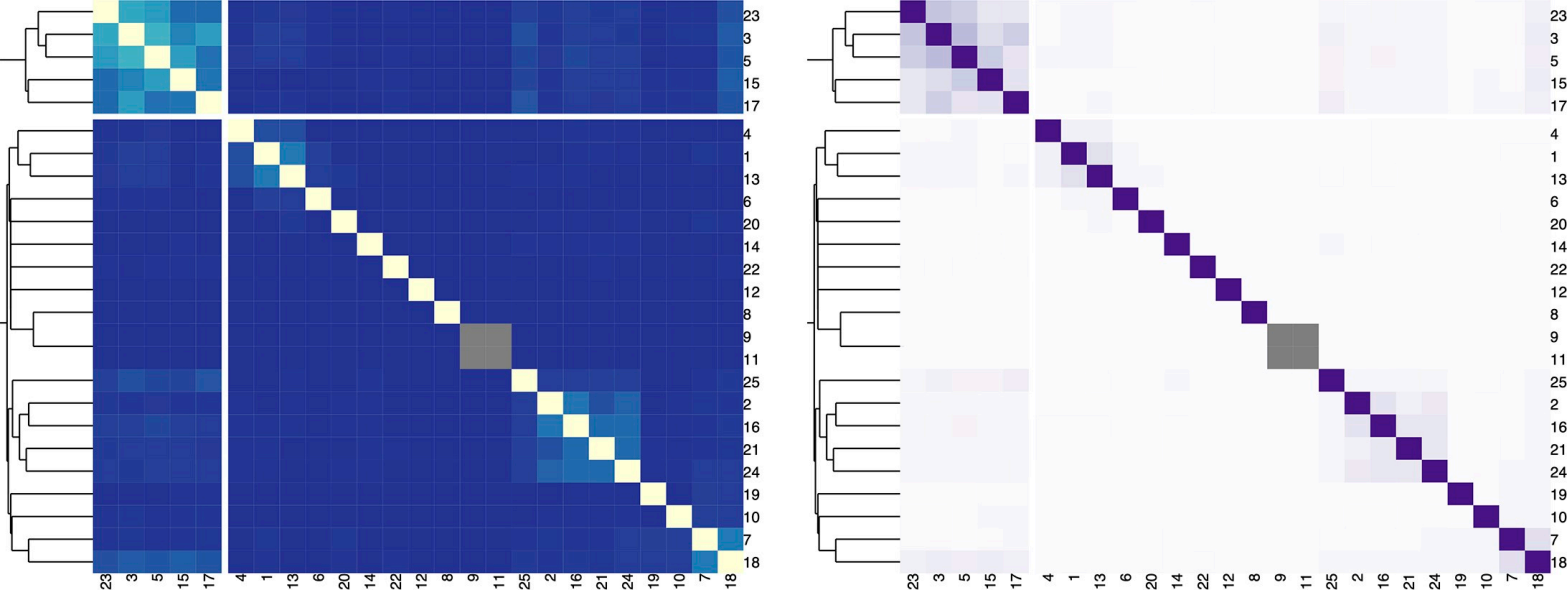
Example of heatmap color mappings. Left: Bad example where a diverging color palette (YlGnBu) is applied to ordered data that progresses from low to high (0 to 1). The color mapping represents higher values in lighter colors. Right: Better example where a sequential color palette (Purples) is applied to the data. The color mapping reverses the importance. Gray cells depict missing data.

For a sequential color palette, the hue should be constrained, and only the luminance or the saturation should vary. It is important to map higher values to darker or lighter colors, depending on the background color, the task at hand, and the nature of the data. In Fig 3, we present a heatmap depicting the Jaccard index among different strings and the contextual information provided by the hierarchical clustering.

For a diverging color palette, it is important when the data have a meaningful or critical break. Typically, the critical break should take a neutral color such as the gray color, and endpoints should take saturated colors. Often symmetrical, the critical break can be the mean, the median, or the zero-change value. In case of the mean or the average, there are often low and high endpoints. In the case of negative and positive values with a zero-value break, the endpoints should use different hues. To accentuate the divergence, the break can be desaturated and the endpoints saturated.

For a qualitative color palette, it is advisable to use only 5–6 colors and only more if absolutely needed [21]. Indeed, when using ColorBrewer, the limit is set within the range from 3 to 9.

## Rule 5: Check for color context in your data vis after the color palette is applied

We perceive colors as constant, if we have reason to, even if they are under different light. Indeed, color constancy is the ability to perceive colors of objects, invariant to the color of the light source. This is mainly due to the fact that color is a relative medium.

**Fig 4:**
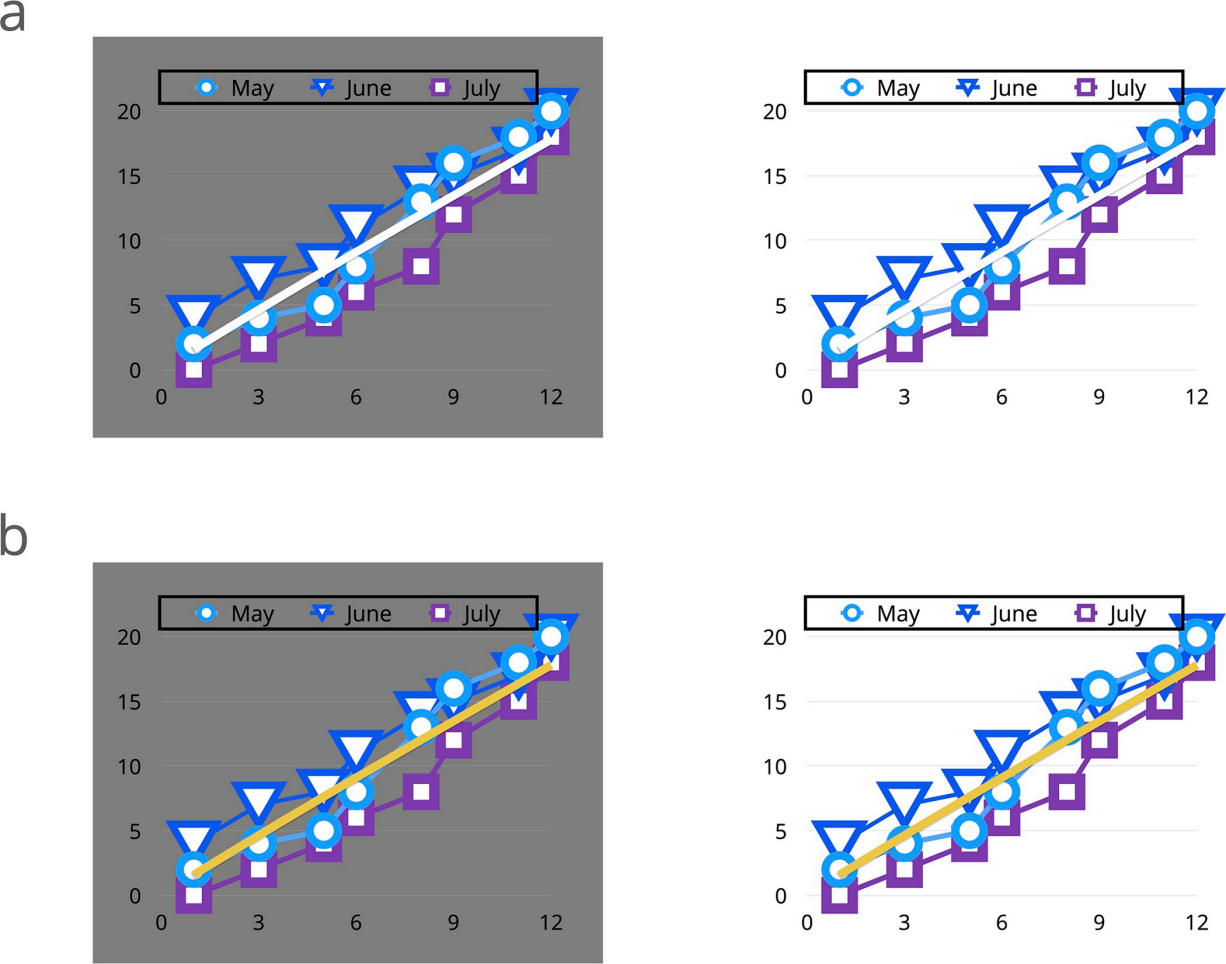
Example of data visualization with a color context-related problem. (a) The example is shown on a computer screen with a gray background versus printed on a paper or shown on a white background. (b) Alternative color encoding of the line using a yellow color solving the problem.

For example, we can see a banana as having a yellow color in the middle of the day with sunshine or in a darkened room with little light. However, there are situations where neighboring colors can alter our perception and ability to distinguish the impact of a certain color. Fig 4 shows an example of data vis where the white can be distinguished against a gray background, perhaps on your computer screen. However, the same white line is difficult to distinguish against a white background, perhaps when printed on a white paper. When the white line is changed to a yellow line, the situation is resolved.

The effect of the context and the investigation of the color constancy phenomenon was extensively investigated by instructors at the Bauhaus in their teaching of fundamental design concepts. Itten (1961) and Albers (1963) later published these principles [22,23]. At the time of writing the present rules, we found no online tools, apps, or software that automatically check for color context in finished visualizations. The “Interaction of Color” app can teach you further about how to be aware of color context, which represents the digital extension of the Interaction of Color book that Josef Albers wrote 50 years ago. It provides exercises for learning about the behavior of color in varying display contexts. The app also allows the creation of personalized color studies and palettes at interactionofcolor.com.

**Fig 5:**
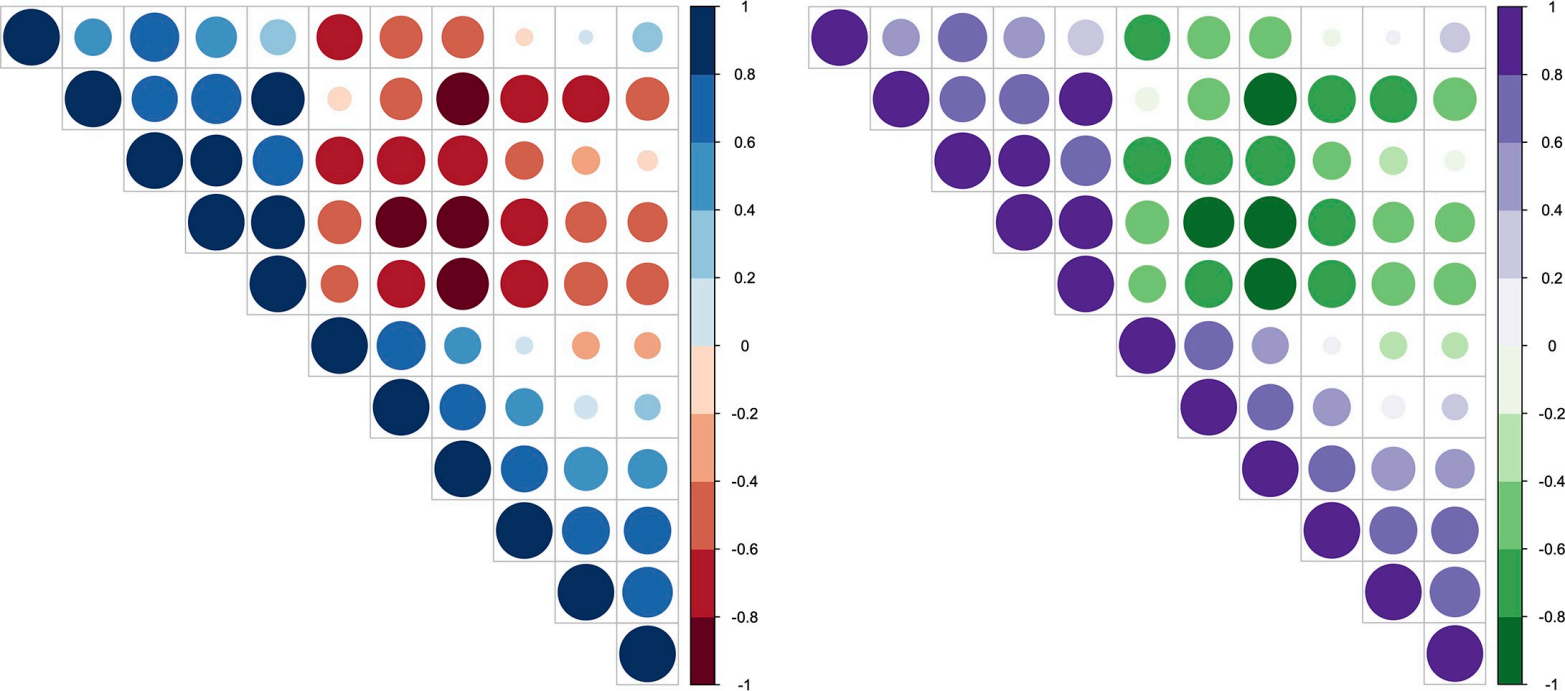
Example of correlation matrix plot with upper triangle. Color intensity and the size of the circle are proportional to the correlation coefficients. Left: Chromatic aberration with the red/blue color combination. Negative/positive correlations: red/blue. Right: Improved data vis with the green/purple complementary color combination. Negative/positive correlations: green/purple.

In biological data vis, it is common to see red/blue-colorized data vis. The preference to a red/blue color combination can be explained, thanks to simultaneous contrast. This may be the case for Fig 5. Simultaneous contrast refers to the way in which 2 different colors affect each other. This is also the reason why it is difficult to read red text on a blue background. The theory is that 1 color can change how we perceive the tone and hue of another when the 2 are placed side by side. The actual colors themselves don't change, but we see them as altered.

The French chemist Michel Eugène Chevreul developed this rule of simultaneous contrast [24]. It maintains that if 2 colors are close together in proximity, each will take on the hue of the complement of the adjacent color. Chevreul’s work “provided the scientific basis for Impressionist and Neo-Impressionist painting,” which included artists such as Eugène Delacroix, Vincent van Gogh, and Robert Delaunay [22]. Just as Chevreul found that 2 simultaneous contrast colors could produce the perception of faded colors in a tapestry after viewing them together for a while, similar results can happen in data visualization. However, it can become difficult to assess the changes in data trends with the use of simultaneous contrasting colors.

## Rule 6: Evaluate interactions of colors in your data visualization

Color usage depends on a lot of data and medium characteristics. Apart from bad interactions of certain colors, we will see that colors may carry meanings, although unintended (c.f., Rule 7). For interactions, there exists a bad interaction of the red/blue colors for textual content. For the reader, the text appears blurred and hurts the eyes. This is the result of a phenomenon called chromatic aberration, which corresponds to a failure to focus on both colors simultaneously. For the provided example in Fig 5, a complementary color combination that also addressed color deficiency issues was desired. The green/purple color scheme provided a contrasting combination between 2 data variables while also allowing individuals with color deficiencies to distinguish between the 2 variables. Green and purple was the optimal combination for this particular situation.

In some cases, uniformity of perception is crucial. A simple example, such as the desire to choose a random color to be readable against a dark background, can be difficult in irregular color spaces because colors of equal brightness or luminosity appear very differently bright (blue and yellow both have 100% brightness in HSV, but blue is much darker than yellow). To counteract such a problem, complicated calculations that take into account the chosen hue are needed to make the random colors appear equally bright. However, there’s an easier way, i.e., choosing a better color space.

**Fig 6:**
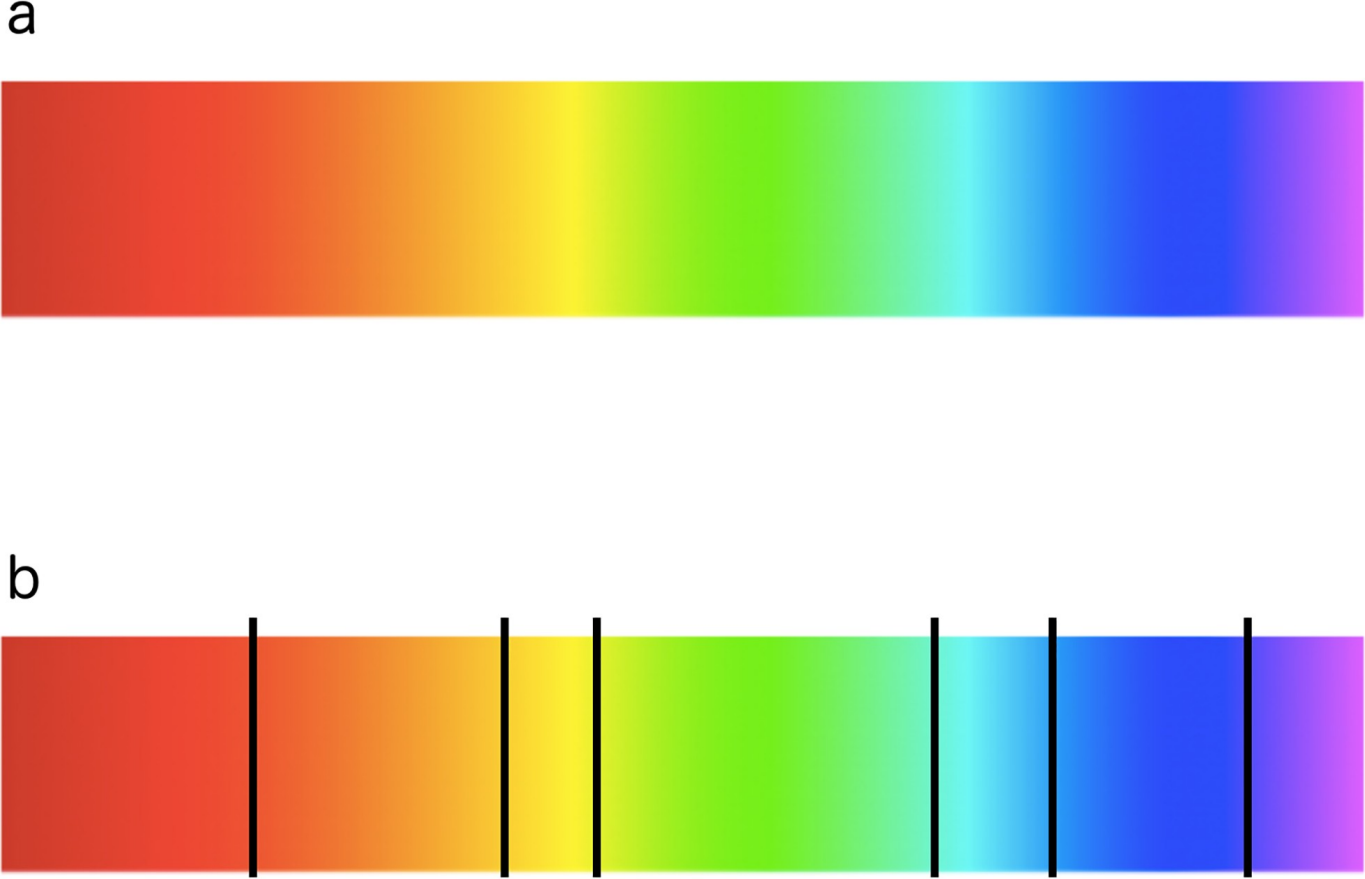
Nonuniform distances between hues in rainbow-based color palettes. (a) Typical rainbow colormap used in visualization tools and analyses. (b) Nonuniform distances between hues. *Image adapted from Applying Color Theory to Digital Media and Visualization*, *p*. *34*, *Problems with the Rainbow Colormap* [12].

The jet or rainbow-based palette is the most commonly used palette as it is delivered as the standard in software tools. It has a high contrast, which makes it capable of accentuating the characteristics of the data at hand. However, when looking at the color chart, bands of color or segments appear especially in the cyan and yellow regions. This seemingly good palette causes sharp transitions when it is applied to depict sequential data with equal importance, although the underlying data varies evenly. Indeed, due to the nonconstant perceptual color changes, it is misleading and even more so for color deficient individuals. Fig 6 depicts these segments. Although many researchers are vocal about its misuse, in many applications, rainbow-based palettes are still employed and have the potential to negatively influence the accuracy at which a task can be solved [25–28].

Unfortunately, since humans generally segment colors into classes, the use of rainbow-like palettes can introduce bias to how the data is interpreted. Moreover, due to the natural order of hues, this may be even amplified. However, different aspects can be integrated intelligently. For example, different lightness emphasizes certain scalar values, while low-luminance colors (e.g., blue) may hide high frequencies [29].

## Rule 7: Be aware of color conventions and definitions in your particular discipline

Describing different levels of biological organization (from molecule through cell, organism, to ecosystem), biology integrates a variety of domains, e.g., biochemistry and biophysics [30]. This involves a multitude of data with different flavors and which may be subject to domain-specific conventions. We briefly discuss 4 notable examples pertaining to biochemistry, biophysics, anatomy, and bacteriology.

First, in chemistry, the colors of the various atoms within a molecule follows the standard Corey Pauling Koltun (CPK) rules [31]. The most important colors are white for hydrogen (H), black for carbon (C), blue for nitrogen (N), red for oxygen (O), deep yellow for sulfur (S), and purple for phosphorus (P). The rest of the atoms follow a light, medium, medium-dark, and dark green for the halogen group and silver for metals [32]. These conventions are followed in biochemistry, for example, to colorize the biochemical structures of the 20 proteinogenic amino acids [33].

Second, in biophysics, a broad range of fluorescent protein genetic variants have been developed over the past several years, featuring fluorescence emission spectral profiles that span almost the entire visible light spectrum. With the help of such molecules and microscopy technologies, scientists can see specific cell responses or even subcellular mechanisms [34,35]. For example, such specific molecules may fluoresce in different spectral light ranges (such as cyan, green, yellow, or red). Admittedly, the most famous molecule is the green fluorescent protein (GFP) [36]. If data sets concern fluorescence profiles, or include information on spectral ranges, the convention is to color the data according to them.

Third, in anatomy, color conventions have existed since the first anatomical sketches. Although the first color-printed medical illustrations have shown literal color usage, modern color usage is rather symbolic [37,38]. Indeed, color is often used for skin tones, internal organs, circulatory and nervous systems, and even selected body tissues (e.g., muscle or fat). Although arteries and nerves appear white, and veins appear whitish blue in vivo, the established color convention is red for arteries, blue for veins, and yellow for nerves [39].

**Fig 7:**
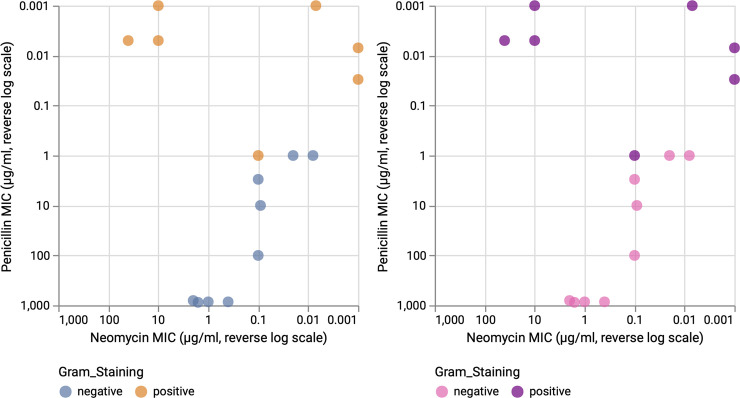
Penicillin and neomycin resistance of bacterial strains. The biological data vis is colorized based on the nominal variable: Gram staining. Left: Domain-independent colors. Right: Domain-dependent colors. These colors better reflect the actual gram staining colors seen under a microscope. The reverse log scale of the MIC is shown for each axis. MIC, minimum inhibitory concentration.

Fourth, in bacteriology, scientists are interested in a multitude of bacterial properties and mechanisms, e.g., gram staining, morphology, genetics, and antibiotic resistance. The former differentiates bacteria by the chemical and physical properties of their cell walls (gram-positive have a thick peptidoglycan cell wall that retains the primary stain of crystal violet) [40]. The latter happens when germs like bacteria and fungi develop the ability to defeat the antibiotics designed to kill them (e.g., penicillin) [41]. Based on data containing the performance of the 3 most popular antibiotics on 16 bacteria, we report 2 example data vis for the effectiveness of the penicillin versus neomycin in Fig 7. While the color scheme blue/orange was chosen to provide perceptually distinguishable colors for nominal comparisons (left), the adoption of the gram staining color convention presents a more adapted and problem-specific color usage (right).

Fifth, other practices exist within specific biological research domains. For example, in molecular and evolutionary biology, the visual encoding of gene expression levels and gene conservation rely on a red/blue diverging palette. However, we cannot speak of a color convention as this varies wildly between red/green, red/blue, and other instances where the break point is not white but, for example, yellow.

A last point deserving mention is to watch out for cultural conventions. Indeed, 1 color may carry very different, if not opposite, symbolic meaning in different countries or cultures. A good example of such an event is the color red, which symbolizes either danger and passion in Western societies or happiness and prosperity in Eastern societies. A study has shown that individuals can come to like or dislike a color according to a localized situation like school rivalries [42]. For instance, University of California, Berkeley (UC Berkeley) students prefer yellow/gold and blue (their school colors) but dislike red and white (Stanford University colors). Color perception is influenced by the long history of competition between the 2 schools in sports and academia. That is why it is important to know your audience.

## Rule 8: Assess color deficiencies

In humans, there are 3 types of photoreceptors or cones where each is sensitive to different parts of the visual spectrum of light to facilitate rich color vision [43,44]. We need to respect that in some humans, color perception is different and assess if the chosen color palette is suitable to individuals with color deficiencies or color blindness. Indeed, if 1 or more of the set of cones does not perform properly, a color deficiency results. A red cone deficiency is classified as **Protanopia**. A green cone deficiency is classified as **Deuteranopia**. A blue cone deficiency is classified as **Tritanopia** [45].

**Fig 8:**
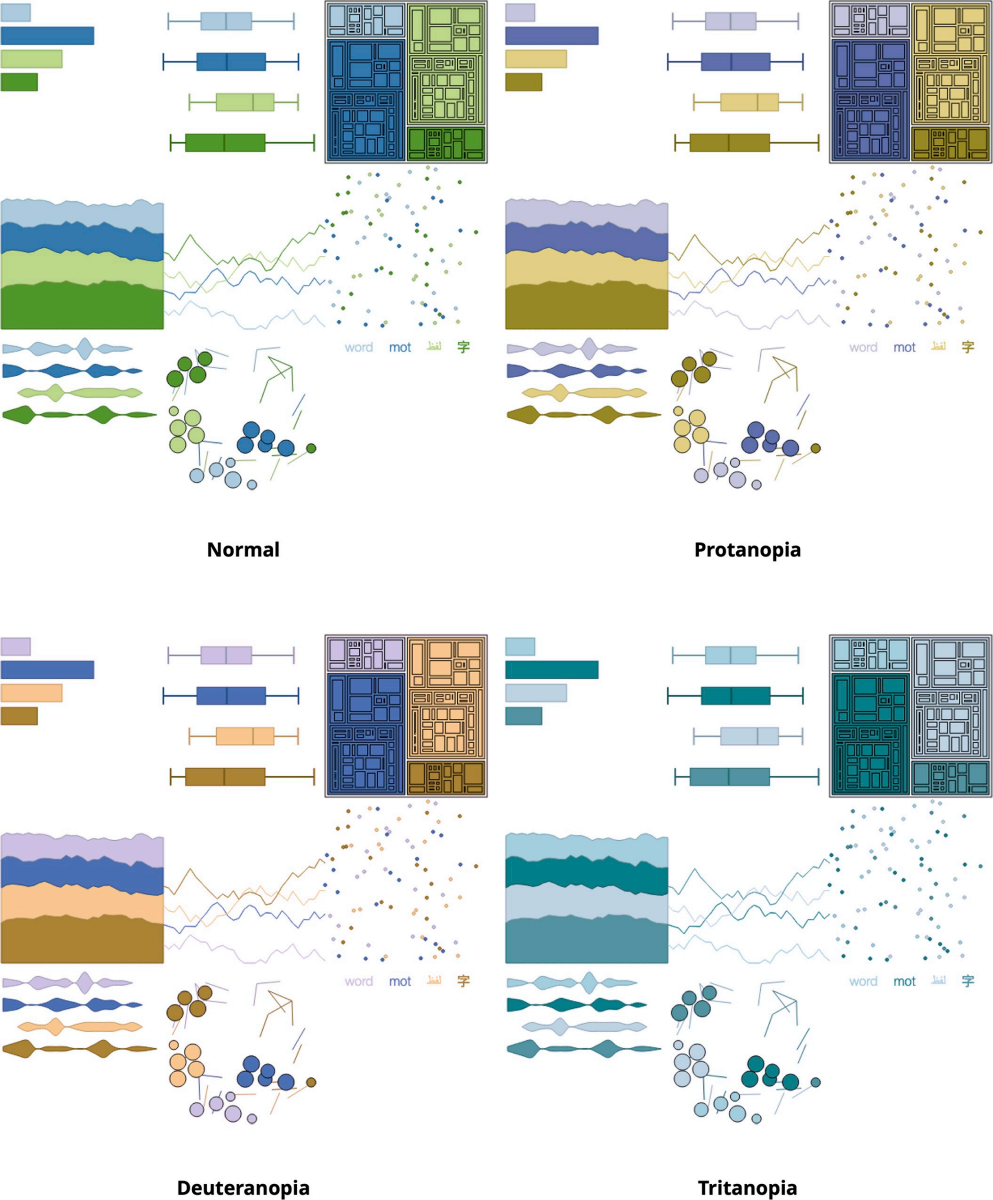
Example test of dichromatic views using Coblis for data vis examples created by Viz Palette. The color palette was created for 4 data classes using ColorBrewer by selecting the qualitative scheme and the color blind safe option. Although the palette is color blind safe, individuals with the very rare tritanopia will have a hard time distinguishing the classes.

When creating or selecting a color palette, different web tools permit testing for color deficiencies and color blindness. On one hand, it’s possible to test color palettes for color deficiencies using either the Adobe Color web tool (color.adobe.com)or the Paletton–The Color Scheme Designer (paletton.com). On the other hand, the web tool Coblis (Matthew Wickline and the Human-Computer Interaction Resource Network) enables us to assess if a data visualization is accessible to a larger audience, including color deficiencies. Coblis is available at color-blindness.com/coblis. Another noteworthy tool is Viz Palette (Elijah Meeks and Susie Lu; https://projects.susielu.com/viz-palette). It allows for testing color deficiencies of specific color palettes by simulating select information visualization examples. Fig 8 combines the 3 tools, ColorBrewer, Viz Palette, and Coblis, to provide an example of a case usage.

## Rule 9: Consider web content accessibility and print realities

There are many situations where biological data visualizations step beyond research efforts and are part of general online (e.g., website) and printed (e.g., journal paper) publications. For these situations, we briefly discuss the web content accessibility and print realities.

For web-based or desktop and mobile devices, we advise following the Web Content Accessibility Guidelines (WCAG), which were created by the World Wide Web Consortium (W3C). There are 12 guidelines organized under 4 principles for which websites must be **perceivable**, **operable**, **understandable**, and **robust**. While there are techniques that help authors meet the guidelines and success criteria, these techniques evolve and are adapted over time. The up-to-date list of techniques can be found at w3.org/TR/WCAG20-TECHS and the guidelines at w3.org/TR/WCAG21. In the listed techniques, there are 8 that concern color (G: 14, 111, 138, 148, 175, 182, 183, 205). We limit the scope to noninteractive data vis and borrow techniques that benefit the accessibility of data visualizations:

- Ensuring that information conveyed by color differences is also available in text (14)
- Using color and pattern (111)
- Using semantic markup whenever color cues are used (138)
- Ensuring that additional visual cues are available when text color differences are used to convey information (182)
- Using a contrast ratio of 3:1 with surrounding text and providing additional visual cues on focus for links or controls where color alone is used to identify them (183)
- Including a text cue for colored form control labels (205).

Indeed, most of these are intended for web page color usage, yet we deem them relevant. The reported techniques address how to improve accessibility for users who cannot see color and hence can look or listen for text cues; people using Braille displays or other tactile interfaces can detect text cues by touch. Additionally, some techniques address the question of achieving a better contrast for textual information and content. Namely, G17: Ensuring that a contrast ratio of at least 7:1 exists between text (and images of text) and background behind the text. Indeed, the idea is to make sure there is a contrast in lightness and not contrast in hue to read the text (c.f., Rule 6: Chromatic aberration). The web tool Colorable (Brent Jackson, Brooklyn, New York, United States) allows the testing of 2 colors using their web Hex code in Hex format and provides slider bars to control hue, saturation, and lightness. It outputs a WCAG contrast ratio as well as a pass/fail decision. The decision ranges from best to worst by relying on the levels of conformance (WCAG): AAA, AA, AA Large, fail. The web tool is available at colorable.jxnblk.com.

Seeing and reading a biological data visualization depends on the medium the target audience uses. On one hand, desktop and mobile devices are used, where a light source is used to mix red, green, and blue in varying intensities in the RGB color space. When all colors are mixed, the white color appears. On the other hand, paper print pieces are used, where a printer combines CMYK colors with varying degrees with physical ink colors: Cyan, magenta, yellow, and black. When all colors are mixed together, the black color results. To facilitate things, we can suggest an easy to follow shortlist listing requirements when working on:

- Desktop and mobile devices, the most suitable color space is RGB. The guiding document in regard to applying color to web pages can be found here: w3.org/TR/css-color-3/#rgb-color
- Small print pieces such as a brochure, or a journal paper figure, we encourage images in CMYK color space at the resolution of 300 DPI
- Very large graphics without always controlling their quality, we advise a conversion from gray scale, bitmap, or RGB color spaces to the printer-friendly CMYK color space.

## Rule 10: Get it right in black and white

Black and white color schemes may be preferred in some situations where printing costs are a concern. Moreover, black and white increases the chance of those who are color blind to see and read your data visualization the same way you do. Seen in different domains, such as graphics and rendering, or even photography, this rule is typically formulated as “Check if it works well in black and white as well as in color.” In data visualization, this often relates to testing if the presented story is still visible or discernible.

Getting it right in black and white can mean 2 things. Either trying your data visualizations in gray scale when uncertain about the color palette or comparing 2 colorized versions of 1 data vis and printing these out in black and white when uncertain which is more readable than the other. Most often, the latter is finding the visualization with the better contrast. Additionally, a noteworthy suggestion concerns photocopy-friendly palettes. To counter the lossy nature of the photocopy process, monochromatic or sequential palettes are the most resilient and suitable.

**Fig 9:**
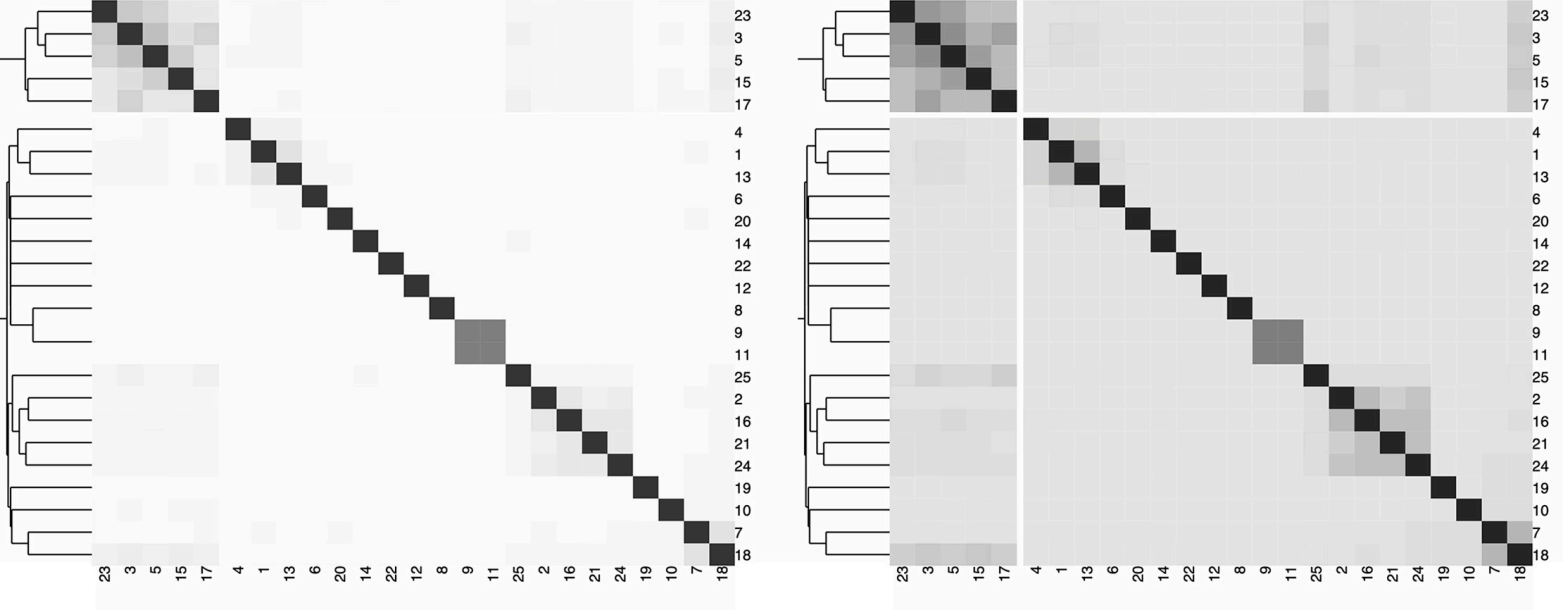
Example of heatmaps adapted from Fig 1. Left: Heatmap obtained by converting Fig 1 (right) to gray scale. If the only relevant information concerns the min and max values, this heatmap is suitable. Right: Midrange values are more visible, thanks to a negatively clipped sequential color palette, i.e., the mapping of the data values is shifted to the darker range of the gray scale. The values of the Jaccard index around 0.5 are brought into the foreground and are more visually pronounced.

**Fig 10:**
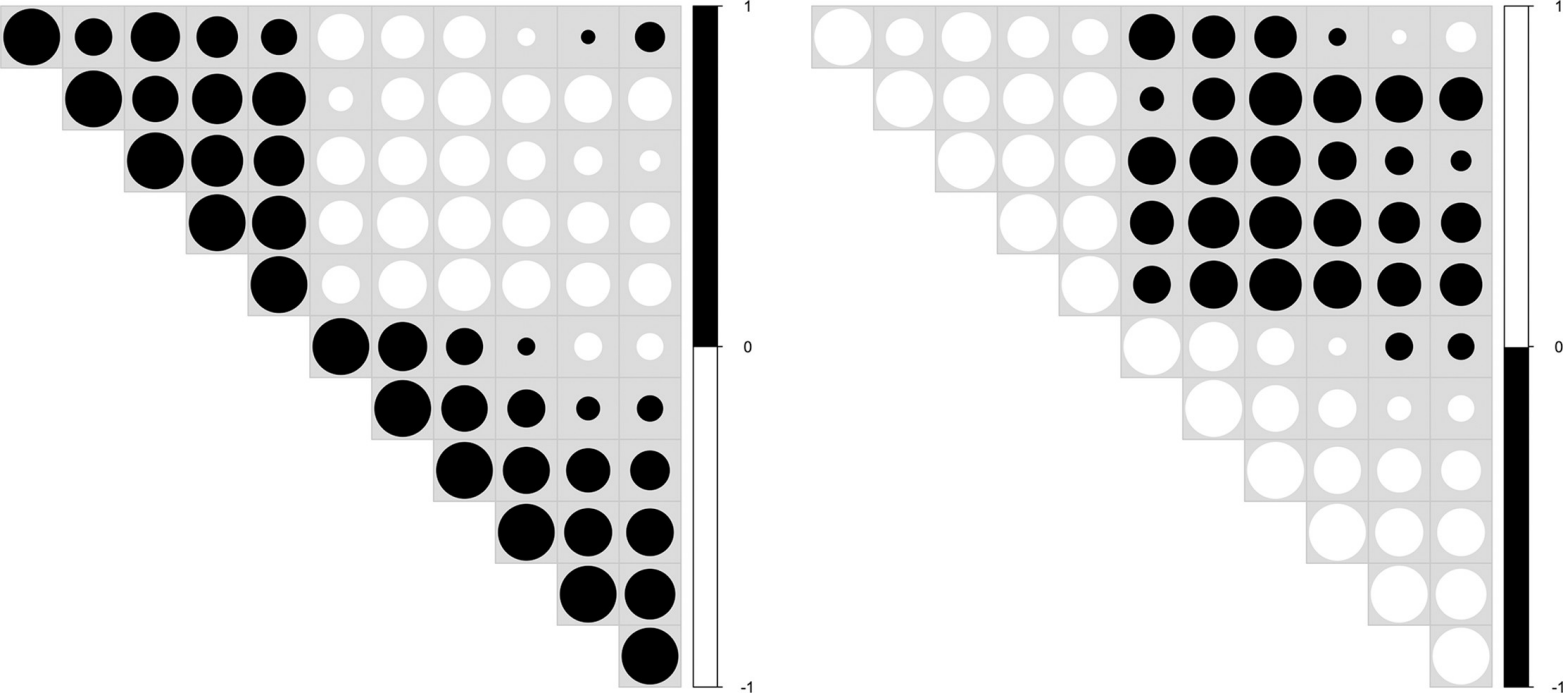
Example of upper triangle adapted from the correlation matrix in Fig 2. Only the size of the circle is proportional to the correlation coefficients. The background is shown in light gray; otherwise, white circles are not visible. Positive/negative correlations are color coded, either in black/white (left) or in white/black (right). Depending on the story we want to convey, the visual importance of positive or negative correlations can be emphasized using black. To limit the focus on 1 specific range of the data, it is also possible to either color code 1 correlation type using a gray scale palette. Since the color of some gray circles may coincide with the background color, one should be aware of such an influence on the audience's perception.

Color is not easy. If there is a need for colors, choose ones that fit and use a few, avoid saturated colors, and be consistent with the audience’s expectations. Figs 9 and 10 detail variations of black and white data visualizations according to example tasks. An example of a safe choice is to select 1 color and several shades of gray. Know your audience and your task better. Above all, do no harm (Primum non nocere).

A summary table can be found at the end of the manuscript for all the discussed rules (c.f., Table 3).

**Table 3:** Summary table describing the purpose of each rule.

Rule	Title	Description
1	Identify the nature of your data	Understanding the data set and the types of variables it contains is important to determine the number of colors and how color should be used
2	Select a color space	Having the right color space ensures that the colors one sees on a monitor or in print will match that of the initial image
3	Create a color palette based on the selected color space	With knowledge of the data, specific rules permit the selection of colors from the chosen color space
4	Apply the color palette to your data set for visualization	Colorizing a data vis adds meaning and it is important to use a color mapping that supports the story behind the data
5	Check for color context in your data vis after the color palette is applied	Surrounding colors may lead to seeing different data points as the same, although their colors are effectively different
6	Evaluate interactions of colors in your data visualization	Understanding which color interactions can be avoided or which ones should be considered helps to design better data visualizations
7	Be aware of color conventions and definitions in your particular discipline	Depending on the audience, some colors may have unintended connotations and meaning; prompting the consideration of other colors
8	Assess color deficiencies	Involving and reaching a much larger audience is an important aspect of visual communication
9	Consider web content accessibility and print realities	Knowing where the final image will be displayed enables the audience to see it as intended
10	Get it right in black and white	Using color sparingly is relevant when color is not needed. Black and white or gray scale visualizations can also be considered
