# Correction: Ten simple rules to colorize biological data visualization

**DOI:** 10.1371/journal.pcbi.1008901

**Published:** 2021-04-06

**Authors:** Georges Hattab, Theresa-Marie Rhyne, Dominik Heider

In Fig 1, the Monochromatic harmony appears twice, so the depiction of the Analogous harmony on the color wheel is erroneous. The authors have provided a corrected version here.

**Fig 1:**
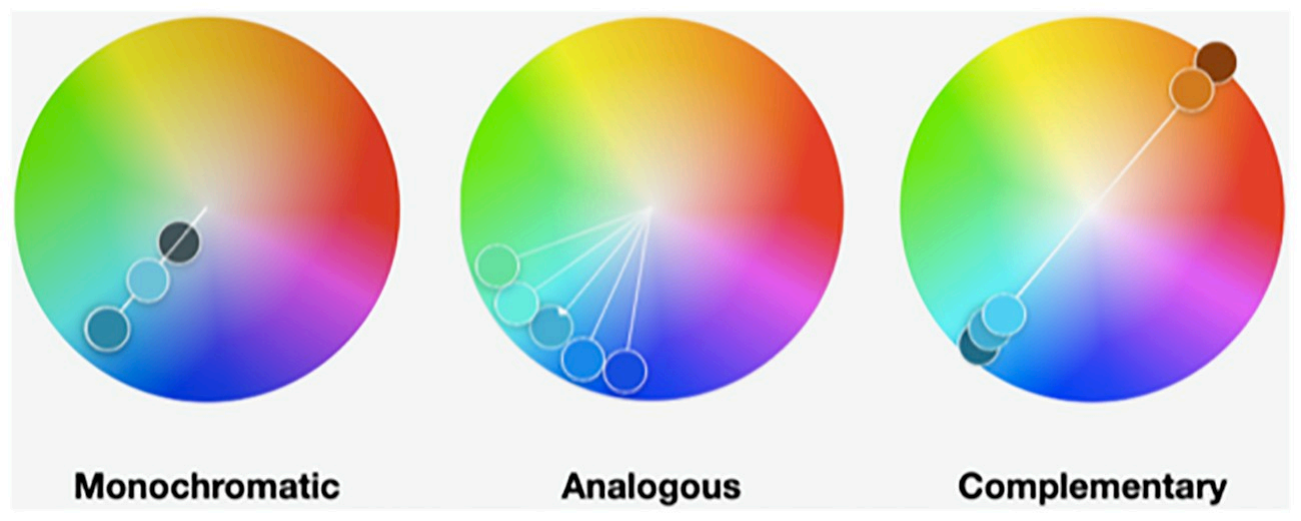
Example of 3 color harmonies in the key of cyan. These harmonies were created using the Adobe Color web tool (color.adobe.com). They are color blind friendly palettes and are presented in Web Hex format. Monochromatic: 2C7C9D, 65BFDA, 39484C. Analogous: 5FE896, 5FF3E3, 3CA7D2, 1E78EF, 1938E3. Complementary: 22607C, 3CA6D0, 4CCFFA, D06D21, 7B3514.
